# Carvacrol Protects IPEC-J2 Cells from Oxidative Stress by Suppressing Autophagy

**DOI:** 10.3390/ijms26083495

**Published:** 2025-04-08

**Authors:** Ming-Chun Hsu, Han-Tsung Wang, Ching-Yi Chen

**Affiliations:** Department of Animal Science and Technology, National Taiwan University, Taipei 10672, Taiwan; a0972956386@gmail.com (M.-C.H.); htwang@ntu.edu.tw (H.-T.W.)

**Keywords:** autophagy, carvacrol, intestinal health, mitochondrial dysfunction, oxidative stress, weaning piglet

## Abstract

Oxidative stress impairs intestinal function and causes poor growth performance in piglets. Carvacrol is a natural essential oil, and its anti-oxidative and anti-inflammatory activities in the intestines of piglets have been reported in many studies. However, the mechanisms underlying these protective effects against oxidative stress remain unclear. This study aimed to investigate the possible pathway of carvacrol in the porcine intestine under oxidative stress using an in vitro model. Porcine intestinal epithelial cells (IPEC-J2) were treated with carvacrol and hydrogen peroxide (H_2_O_2_), an oxidative stress inducer, to investigate the protective mechanisms of carvacrol under oxidative stress. We found that carvacrol ameliorated a H_2_O_2_-induced loss of cell viability, apoptosis, and reduced intracellular reactive oxygen species (ROS) and malondialdehyde (MDA) levels. Carvacrol reduced mitochondrial ROS generation and increased citrate synthase activity during oxidative stress. Furthermore, carvacrol attenuated an increase in the autophagy marker LC3II-to-I ratio and reduced the accumulation of lysosomes and autolysosomes induced by H_2_O_2_. The increased protein expression of the mitophagy marker PINK1, induced by H_2_O_2_, was also reduced by carvacrol treatment. Metformin-activated autophagy diminished the protective effects of carvacrol on cell viability and MDA levels under H_2_O_2_ treatment, indicating that autophagy inhibition is necessary for carvacrol-induced protection in IPEC-J2 cells during oxidative stress. In conclusion, this study demonstrated the underlying mechanism that carvacrol exerted its anti-oxidative effects on porcine intestinal epithelial cells by relieving excessive autophagy during weaning stress.

## 1. Introduction

Oxidative stress results from an imbalance between pro-oxidants and anti-oxidants. On commercial pig farms, pigs usually face many stressful events, such as early weaning, disease, and environmental factors, which disturb redox homeostasis and cause intestinal oxidative stress. The intestine is the most important organ involved in feed digestion and nutrient absorption in animals [1]. These factors may cause oxidative damage to the intestinal structure, microbial dysbiosis, and impaired nutrient absorption, which leads to poor growth performance in pigs and has a disastrous economic impact on pig farms.

The metabolic functions of mitochondria play an important role in maintaining multiple functions of the intestinal epithelium, such as cell renewal, barrier function, nutrient digestion, and absorption [2]. However, mitochondrial dysfunction leads to reactive oxygen species (ROS) generation, which is associated with mitochondria-dependent apoptosis. Moreover, ROS-induced necroptosis leads to mitochondrial DNA release as damage-associated molecular patterns that trigger intestinal inflammation [3]. To maintain a healthy mitochondrial pool in the intestine, the quality of mitochondria can be tightly regulated via selective autophagy and mitophagy [4]. Autophagy selectively degrades and recycles unnecessary or damaged substances to maintain cellular homeostasis [5]. Macroautophagy, often referred to as autophagy, is the best-known type of autophagy. Depending on the different cargos, there are several subtypes of autophagy, such as nucleophagy, lipophagy, and mitophagy, which are responsible for the clearance of damaged nuclear materials, lipid droplets, and abnormal mitochondria, respectively [6]. Mitophagy is a mechanism that is responsible for clearing abnormal mitochondria. During mitophagy activation, PINK1 acts as a sensor of damaged or dysfunctional mitochondria and then recruits PARKIN to the outer mitochondrial membrane. The activation of PARKIN facilitates the formation of polyubiquitinated protein chains on the mitochondrial surface, which amplify mitophagy signals, and these polyubiquitinated protein chains are targeted by the autophagic receptors. Via directly interacting with LC3II, these defective mitochondria are engulfed by the double-membrane vesicles, forming autophagosomes, for further degradation with lysosomes [7]. Recent studies have confirmed the importance of a healthy mitochondrial pool in maintaining intestinal function [8,9]. In addition, mitochondrial dysfunction has been observed in the intestine of piglets during weaning-induced oxidative stress [10,11], indicating the potential of mitochondria as a therapeutic target during intestinal oxidative stress.

Carvacrol is a phenolic monoterpene compound naturally found in thyme, oregano, wild bergamot, pepperwort, and other plants. Several biological activities of carvacrol have been studied, including anti-microbial, anti-oxidative, anti-inflammatory, and anti-cancer effects [12,13]. Due to the multiple beneficial bioactivities of carvacrol and the development of anti-microbial resistance due to the misuse of antibiotics, carvacrol is a promising feed additive to replace antibiotic growth promoters in the farm industry [14]. Recent studies have demonstrated the anti-oxidative and anti-inflammatory effects of carvacrol-containing diets on the intestines of weaning piglets [15,16]. However, the exact molecular mechanisms underlying the action of carvacrol on intestinal cells remain unclear.

This study aimed to elucidate the possible mechanisms of action of carvacrol in mitochondrial function, autophagy, and mitophagy using an in vitro model of intestinal oxidative stress in porcine intestinal epithelial cells (IPEC-J2).

## 2. Results

### 2.1. Carvacrol Reduced H_2_O_2_-Induced Apoptosis in IPEC-J2 Cells

**Figure 1:**
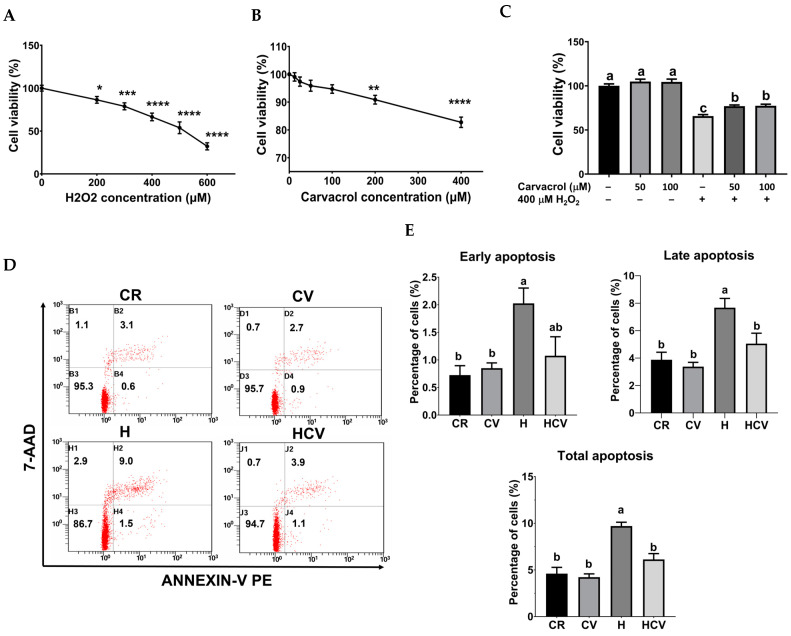
Carvacrol mitigated H_2_O_2_-induced IPEC J2 death. **The** cytotoxicity of H_2_O_2_ (**A**) and carvacrol (**B**) was determined by treating with H_2_O_2_ or carvacrol for 24 h, respectively. To study the protective effect of carvacrol against H_2_O_2_-induced death, cells were pretreated with carvacrol for 6 h and then incubated with 400 μM H_2_O_2_ for 24 h without changing the medium. Cell viability (**C**) was determined by a MTT assay. Apoptosis was analyzed by flow cytometry (**D**) and quantitated (**E**). Values were represented as means ± SEM, n = 4. * *p* < 0.05, ** *p* < 0.01, *** *p* < 0.001, and **** *p* < 0.0001, compared with untreated control cells. ^abc^ Means without a common letter differ, *p* < 0.05. CR: control with 0.05% DMSO, CV: 50 μM carvacrol, H: 400 μM H_2_O_2_, HCV: 50 μM carvacrol +400 μM H_2_O_2_.

To investigate the protective effects of carvacrol against oxidative stress-induced intestinal damage in piglets, an in vitro model was established using IPEC-J2 cells following H_2_O_2_ treatment. Treatment with 200 to 600 μM H_2_O_2_ for 24 h led to a dose-dependent decrease in cell viability (Figure 1A), with 400 μM yielding approximately 65% viability compared to untreated cells, making it the chosen concentration for further assessments. To assess carvacrol’s cytotoxicity, IPEC-J2 cells were incubated with 12.5 to 400 μM of carvacrol for 24 h. Results showed that concentrations below 200 μM did not significantly affect cell viability (Figure 1B), indicating they were non-toxic. Therefore, amounts of 50 and 100 μM carvacrol were selected for further studies.

The cytoprotective role of carvacrol against oxidative stress was determined using an MTT assay. IPEC-J2 cells were pretreated with 50 or 100 μM carvacrol for 6 h, followed by exposure to 400 μM H_2_O_2_ for an additional 24 h without changing the medium. As shown in Figure 1C, both concentrations of carvacrol significantly increased cell viability compared to the H_2_O_2_-treated group in the absence of carvacrol (*p* < 0.05), indicating the cytoprotective ability of carvacrol against oxidative stress in IPEC-J2 cells [17]. Therefore, 50 μM carvacrol was used for subsequent assessments.

Since apoptosis, is closely related to oxidative stress-induced cell death, apoptotic cells were determined using Annexin-V and 7-AAD staining. As shown in Figure 1D,E, 400 μM H_2_O_2_ treatment (H) for 24 h significantly increased the percentage of both early and late apoptotic cells. In contrast, 50 μM carvacrol pretreatment (HCV) for 6 h significantly decreased the percentage of late and total apoptotic cells (*p* < 0.05), indicating carvacrol prevented H_2_O_2_-induced apoptosis in IPEC-J2 cells.

### 2.2. Carvacrol Relieved H_2_O_2_-Induced Oxidative Stress in IPEC-J2 Cells

**Figure 2:**
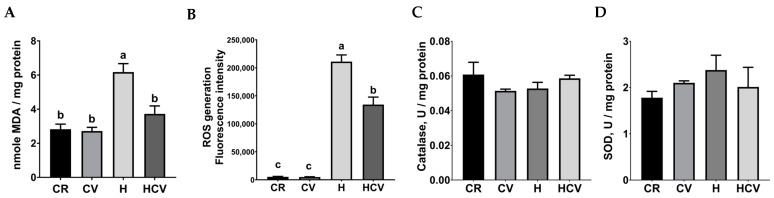
Carvacrol attenuated H_2_O_2_-induced oxidative stress in IPEC J2 cells. Oxidative products (**A**), lipid peroxidation; (**B**), ROS generation and anti-oxidative enzyme activities (**C**), catalase; (**D**), SOD) were determined. Cells were pretreated with carvacrol for 6 h and then incubated with 400 μM H_2_O_2_ for 6 h without changing the medium. Values were represented as means ± SEM, n = 3. ^abc^ Means without a common letter differ, *p* < 0.05. CR: control with 0.05% DMSO, CV: 50 μM carvacrol, H: 400 μM H_2_O_2_, HCV: 50 μM carvacrol +400 μM H_2_O_2._.

Previous studies have shown the protective effects of carvacrol against oxidative stress in various cells [18,19,20]; however, whether carvacrol retains its anti-oxidative role in intestinal cells remains unclear. Consequently, markers of oxidative and anti-oxidative systems were investigated. The intracellular MDA level was determined as a marker of lipid peroxidation. As shown in Figure 2A, compared to the control group, 400 μM H_2_O_2_ treatment (H) significantly increased the MDA level (*p* < 0.05). In contrast, 50 μM carvacrol pretreatment (HCV) significantly decreased the MDA level compared to the H_2_O_2_ treatment group (*p* < 0.05). Intracellular ROS generation was determined by DCFDA staining. As shown in Figure 2B, 50 μM carvacrol pretreatment (HCV) significantly decreased the ROS level induced by the H_2_O_2_ treatment (*p* < 0.05). As the important enzymes of the innate anti-oxidative system, the activities of catalase and superoxide dismutase (SOD) were also determined. As shown in Figure 2C,D, neither carvacrol nor H_2_O_2_ significantly affected the activities of catalase and SOD (*p* > 0.05). These results indicated that carvacrol protected against oxidative stress by alleviating oxidative reactions rather than by activating anti-oxidative enzymes.

### 2.3. Carvacrol Ameliorated Mitochondrial Dysfunction in IPEC-J2 Cells Under Oxidative Stress

**Figure 3:**
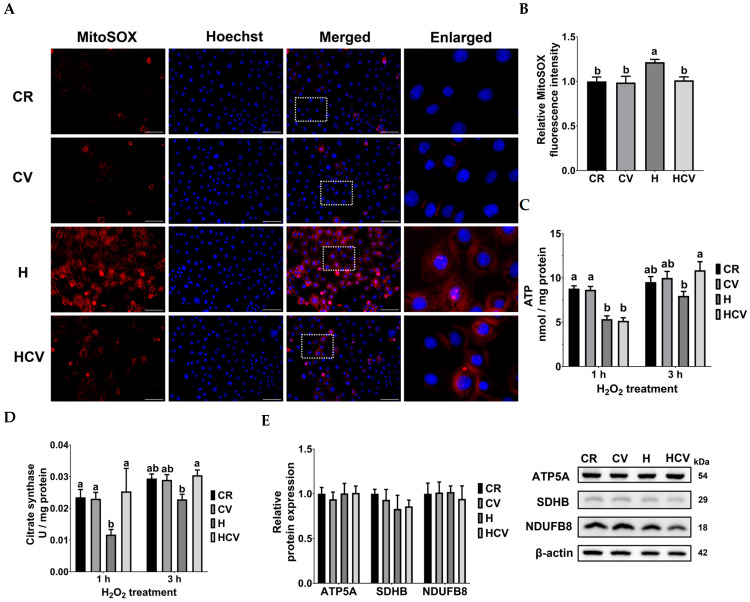
Carvacrol reduced H_2_O_2_-induced mitochondrial dysfunctions in IPEC J2 cells. (**A**,**B**) mitochondrial ROS were detected by MitoSOX (red fluorescence) and nucleoli were stained with Hoechst (blue fluorescence). (**A**) Representative images of mitochondrial ROS generation after carvacrol and H_2_O_2_ treatment. The white box is enlarged, as shown in the picture on the right panel. Scale bar: 100 μm. (**B**) Quantitative analysis of MitoSOX red intensity after carvacrol and H_2_O_2_ treatment. (**C**) Intracellular ATP level, (**D**) citrate synthase activity, and (**E**) OXPHOS-related protein expressions. Cells were pretreated with carvacrol for 6 h and then incubated with 400 μM H_2_O_2_ for 3 h without changing the medium. Values were represented as means ± SEM, n = 5. ^ab^ Means without a common letter differ, *p* < 0.05. CR: control with 0.05% DMSO, CV: 50 μM carvacrol, H: 400 μM H_2_O_2_, HCV: 50 μM carvacrol +400 μM H_2_O_2_.

During oxidative stress, mitochondria are among the most vulnerable organelles, and their dysfunction results in excess mitochondrial ROS generation, which aggravates oxidative stress in cells. As shown in Figure 3A, mitochondrial ROS were visualized with mitoSOX staining using red fluorescence. Fluorescence imaging revealed an obvious increase in red fluorescence around the nucleoli stained with Hoechst during H_2_O_2_ treatment. Quantitative results showed that compared to the CR group, H_2_O_2_ treatment (H) significantly increased mitochondrial ROS production (*p* < 0.05). In contrast, carvacrol pretreatment (HCV) significantly decreased mitochondrial ROS production compared to that in the H_2_O_2_ treatment group (*p* < 0.05) (Figure 3B).

An increase in mitochondrial ROS generation can lead to mitochondrial dysfunction in energy metabolism. Intracellular ATP levels, citrate synthase activity, and OXPHOS complexes-related protein expression were estimated to evaluate mitochondrial function. As shown in Figure 3C, treatment with H_2_O_2_ for 1 h significantly reduced intracellular ATP levels compared to those in the CR group (*p* < 0.05). However, it took an additional 2 h for carvacrol to restore ATP levels that were diminished by H_2_O_2_ treatment (*p* > 0.05).

The production of NADH and FADH_2_ from the TCA cycle is indispensable for energy generation in mitochondrial OXPHOS. Citrate synthase, which utilizes acetyl-CoA and oxaloacetate to generate citrate, is a key enzyme in the TCA cycle. As shown in Figure 3D, citrate synthase activity was inhibited when cells were exposed to H_2_O_2_ for 1 h, while carvacrol pretreatment simultaneously recovered enzyme activity. The protein expression of the subunits of OXPHOS complexes was also estimated to evaluate the mitochondrial OXPHOS function. Results showed that there were no significant differences in the protein expression of ATP5A (complex V), SDHB (complex II), or NDUFB8 (complex I) among the groups (Figure 3E). These results indicated that carvacrol activated citrate synthase activity, thereby restoring ATP production in IPEC J2 cells under oxidative stress. Interestingly, there was a time lag between citrate synthase and ATP levels by carvacrol regulation, which might be because the recovery of mitochondrial OXPHOS for ATP generation requires more time.

### 2.4. Carvacrol Inhibited Mitophagy in IPEC-J2 Cells Under Oxidative Stress

**Figure 4:**
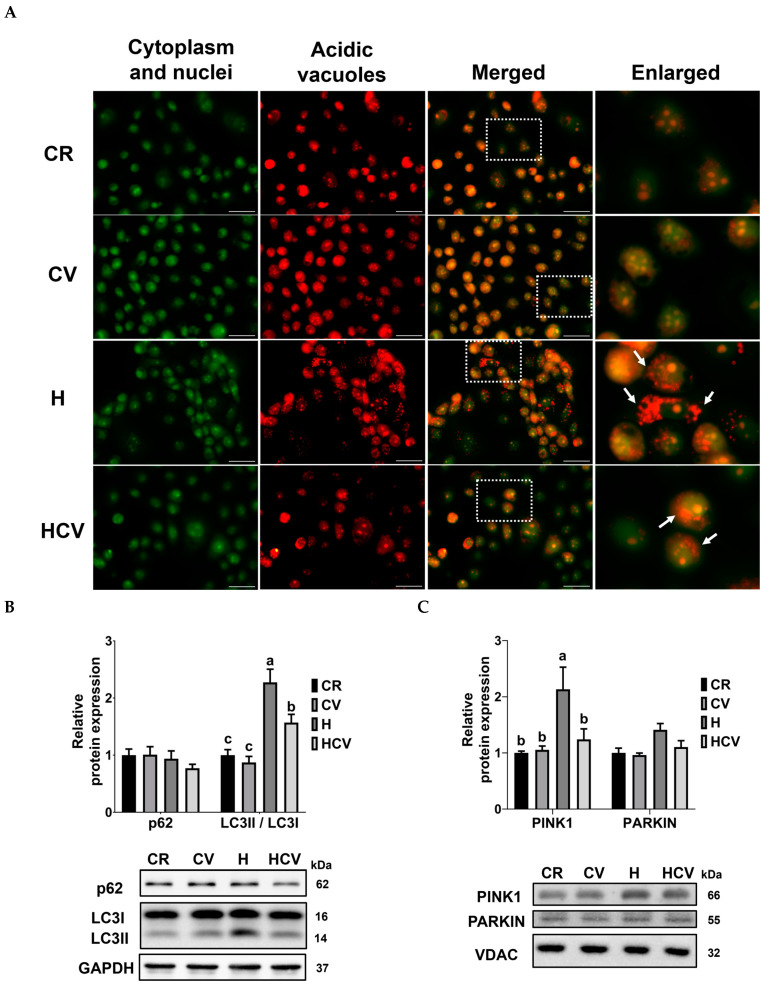
Carvacrol suppressed H_2_O_2_-induced mitophagy in IPEC J2 cells. (**A**) Acidic vacuoles, (**B**) autophagy-related protein expressions, and (**C**) mitophagy-related protein expressions. Cells were pretreated with 50 μM carvacrol for 6 h and then incubated with 400 μM H_2_O_2_ for 3 h without changing the medium. Acridine orange staining was used to detect the acidic vacuoles (red fluorescence) as the marker of autophagy. Green fluorescence represents the cytoplasm and nucleolus. White arrows: acidic vacuoles. The white box is enlarged, as shown in the picture on the right panel. Scale bar: 50 μm. The expressions of VDAC and GAPDH were used as the internal control, respectively. Values were represented as means ± SEM, n = 4–6. ^abc^ Means without a common letter differ, *p* < 0.05. CR: control with 0.05% DMSO, CV: 50 μM carvacrol, H: 400 μM H_2_O_2_, HCV: 50 μM carvacrol + 400 μM H_2_O_2_.

Because oxidative stress and mitochondrial dysfunction may activate autophagy, acidic vacuoles and proteins related to autophagy and mitophagy were assessed. In Figure 4A, the accumulation of acidic vacuoles, including lysosomes and autolysosomes, was visualized with acridine orange staining to evaluate autophagy. Representative images show that H_2_O_2_ treatment induces the accumulation of red fluorescent puncta (white arrows), lysosomes, or autolysosomes in the cytoplasm. Furthermore, pretreatment with carvacrol effectively prevented the accumulation of acidic vacuoles. The autophagy pathway-related proteins p62 and LC3 were assessed using Western blotting. As shown in Figure 4B, there were no significant differences in p62 expression among the groups. The ratio of LC3II to I was significantly increased by H_2_O_2_ treatment (*p* < 0.05). In contrast, carvacrol pretreatment decreased the LC3II-to-I ratio after H_2_O_2_ treatment (*p* < 0.05). Mitophagy plays a crucial role in removing dysfunctional mitochondria. Consequently, we evaluated the expression of the mitophagy-related proteins, PINK1 and PARKIN, using Western blotting. As shown in Figure 4C, H_2_O_2_ treatment increased the expression of PINK1, which was effectively reversed by carvacrol pretreatment (*p* < 0.05). Regarding the expression of PARKIN, no significant differences were observed among the groups (*p* > 0.05). These results supported that carvacrol attenuated H_2_O_2_-induced mitophagy in IPEC J2 cells.

### 2.5. Autophagy Inhibition Was Necessary for Carvacrol’s Protection in IPEC-J2 Cells Under Oxidative Stress

**Figure 5:**
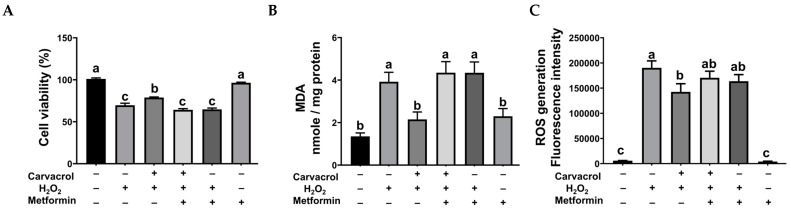
Inhibiting autophagy is necessary for carvacrol’s protection in IPEC-J2 cells exposed to oxidative stress. (**A**) cell viability, (**B**) MDA level, and (**C**) ROS generation. Cells were pretreated with 50 μM carvacrol for 6 h and then incubated with 400 μM H_2_O_2_ for the indicated duration (24 h for cell viability and 6 h for MDA and ROS generation). One hour prior to the treatment of H_2_O_2_, 500 μM metformin was added to induce autophagy. Values were represented as means ± SEM, n = 3–6. ^abc^ Means without a common letter differ, *p* < 0.05.

Metformin, a known autophagy activator, was used to elucidate the role of autophagy in the protective mechanism of carvacrol against oxidative stress. As shown in Figure 5, autophagy activation by metformin significantly decreased cell viability and increased MDA levels after carvacrol and H_2_O_2_ treatments (*p* < 0.05). However, ROS generation was not affected by metformin. These results indicated that autophagy activation diminished the protective effects of carvacrol in IPEC-J2 cells during H_2_O_2_-induced oxidative stress.

## 3. Discussion

Carvacrol exhibits various biological activities, including anti-microbial, anti-oxidative, anti-inflammatory, and anti-cancer effects [13]. Recent studies have shown that carvacrol-containing diets enhance intestinal health and growth performance of weaning piglets [15,16,21,22]. This study investigated the protective effect of carvacrol on intestine health during weaning stress, utilizing an in vitro model with a particular focus on mitochondrial functions. Our findings revealed carvacrol’s regulation of mitochondrial clearance (autophagic pathway) in IPEC-J2 cells during oxidative stress [17].

Apoptosis is believed to contribute to intestinal injury during severe oxidative stress [23]. Wang et al. [24] indicated that H_2_O_2_ caused caspase-3 activation and induced apoptosis in rat intestinal epithelial cells. Also, Zhang et al. [25] demonstrated that H_2_O_2_ increased the protein expression of BAX and decreased the protein expression of BCL2, with elevated numbers of apoptotic cells in ovine intestinal epithelial cells. Moreover, apoptosis plays an important role in early weaning-induced intestinal oxidative stress. Zhu et al. [26] revealed that apoptosis-related genes were differentially expressed in the jejunum of piglets after weaning. Furthermore, Tang et al. [27] showed caspase-1 and 3 activation and an increased number of apoptotic cells in the colon tissue of piglets after weaning. In the current study, we demonstrated that H_2_O_2_ treatment significantly increased the number of apoptotic cells, indicating the activation of apoptosis during oxidative stress in porcine intestinal epithelial cells. These results are in line with previous studies [28,29]. Moreover, we found that carvacrol pretreatment significantly reduced the number of apoptotic IPEC-J2 cells exposed to H_2_O_2_, indicating the capacity of carvacrol to attenuate apoptosis during intestinal oxidative stress in weaning piglets. Moreover, H_2_O_2_-induced apoptosis may be associated with the intrinsic pathway via mitochondrial dysfunction [30]. These results revealed that carvacrol effectively attenuated cell death under H_2_O_2_-induced oxidative stress in porcine intestinal epithelial cells.

The gastrointestinal tract serves as the first line of contact with the outer environment and is responsible for barrier function, preventing the invasion of foreign substances, which are among the most important sources of ROS. ROS directly attacks proteins, lipids, and DNA, which are the basic components of cells, and therefore, ROS impairs the intestinal structure and function [31]. During intestinal oxidative stress, the redox status may be disturbed, including an increase in oxidants or pro-oxidants, MDA and ROS levels, and decreased activities of anti-oxidative enzymes, SOD, catalase, and glutathione peroxidase. Previous studies have reported the chemical anti-oxidant properties of carvacrol due to its resonance structure, which stabilizes free radicals [13]. In addition, Zou et al. [32] indicated that the anti-oxidative capacity of oregano essential oil (containing 80% carvacrol) may result from Nrf2 activation, which promotes the gene expression of anti-oxidative enzymes; similar effects of carvacrol have also been shown in several studies [33,34]. Consistently, our previous in vivo study demonstrated that using an essential oil mixture (containing 200 ppm of carvacrol) for 4 weeks effectively increased total anti-oxidative capacity and reduced lipid peroxidation in the plasma of both weaning piglets and growing pigs [22]. In the current study, we demonstrated that carvacrol reversed the adverse effects of H_2_O_2_ treatment, including cell viability and ROS and MDA levels, in IPEC J2 cells, indicating the protective and anti-oxidative capacities of carvacrol on intestinal epithelial cells under oxidative stress. These results are in line with the study by Zou et al. [32], who demonstrated that oregano essential oil protected IPEC-J2 cells from H_2_O_2_-induced oxidative stress and cell death.

Mitochondria are not only the main target of oxidative stress, but also the main producers of ROS, and mitochondrial dysfunction has been reported in intestinal diseases in several studies [3,4,35]. Our results demonstrated that carvacrol pretreatment effectively reduced mitochondrial ROS levels and increased citrate synthase activity and ATP levels during oxidative stress. These results are consistent with those of Chenet et al. [19], which demonstrated the protective effect of carvacrol on mitochondrial function in human neuroblastoma cells. The mitochondrial protective effects of carvacrol may explain its ability to reduce apoptosis by attenuating the activation of mitochondria-dependent intrinsic pathways [36]. However, our results showed that the decline in ATP levels during oxidative stress was not ameliorated by carvacrol until 3 h later, indicating that although citrate synthase activity was elevated to provide substrates, the function of mitochondrial OXPHOS in ATP generation might require more recovery time.

Macroautophagy, simply referred to as autophagy, and its subtype mitophagy, which is specific for abnormal mitochondrial clearance, are cell-protective mechanisms that maintain intracellular homeostasis during stress conditions [37]. However, excessive autophagy may result in excessive consumption of cytoplasmic components, such as proteins, macromolecules, mitochondria, or other organelles, further leading to cell death, which is considered autophagy-dependent [38]. Liao et al. [39] indicated that the inhibition of autophagy by chloroquine significantly improved intestinal morphology and increased tight junction-related mRNA expression with increasing growth performance in weaning piglets, suggesting that autophagy-dependent cell death is involved in intestinal injury during weaning stress. Furthermore, oxidative stress-induced autophagy-dependent cell death has been proved in H_2_O_2_-induced human glioblastoma and embryonic kidney cells, which showed increased cell viability during autophagy inhibition [40]. Our results demonstrated that carvacrol pretreatment effectively reduced the accumulation of acidic vacuoles and the LC3II-to-I ratio, and significantly downregulated PINK1 at the protein level, indicating that carvacrol might inhibit excessive autophagy and mitophagy in intestinal epithelial cells during oxidative stress.

In previous studies, Simsek et al. [20] demonstrated that carvacrol inhibited autophagy activation, including the protein and mRNA expression of Beclin-1 and LC3II, during mercuric chloride-induced toxicity in rat testicular tissue, and similar effects were also shown by Spalletta et al. [41], who demonstrated that carvacrol suppressed adipogenic differentiation via autophagy inhibition in murine preadipocytes. Conversely, Arruri et al. [42] demonstrated that carvacrol recovered impaired autophagy during peripheral neuropathic pain in rat sciatic nerves. These studies indicate that the modulation of autophagy by carvacrol may depend on the cells, tissues, and stress conditions.

**Figure 6:**
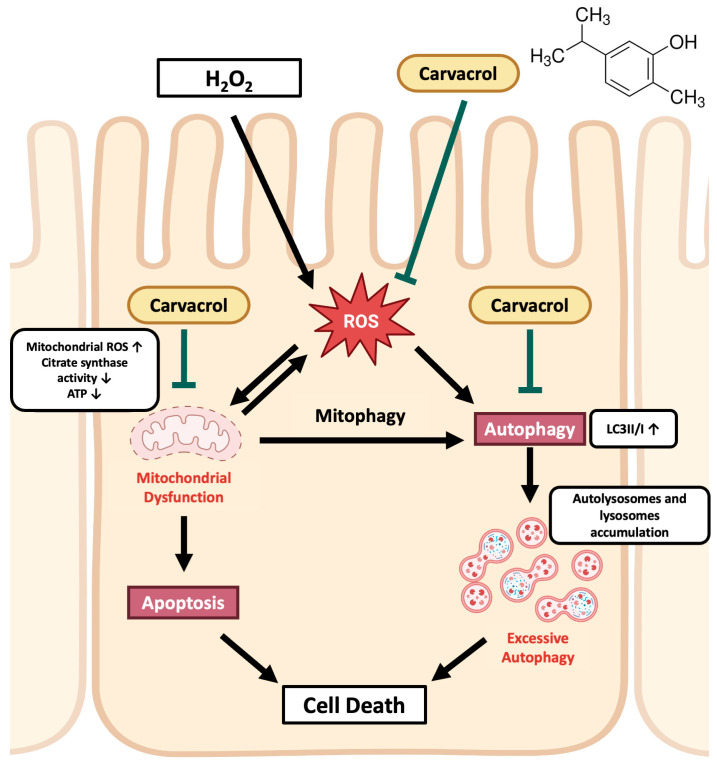
The model of carvacrol’s protective effects on IPEC-J2 cells during H_2_O_2_-induced oxidative damages. Carvacrol protected IPEC-J2 cells from oxidative damage, which resulted from the inhibition of oxidative stress and excessive autophagy and might be related to mitochondrial protection to attenuate apoptosis.

To elucidate the role of carvacrol in regulating autophagy during intestinal oxidative stress, we used metformin as an autophagy activator in our in vitro model. Metformin, an AMPK activator, is a well-known drug that has long been used for the treatment of type II diabetes [43]. Due to its regulation of AMPK, the ability of metformin to activate autophagy has also been validated in several studies [5,37,44]. Our results demonstrated that autophagy activation by metformin significantly diminished the protective effects of carvacrol on cell viability and MDA levels during H_2_O_2_-induced oxidative damage, indicating that autophagy suppression by carvacrol is necessary for cell survival and for relieving lipid peroxidation during oxidative stress in porcine intestinal epithelial cells. To the best of our knowledge, this is the first study to demonstrate that carvacrol suppressed autophagy in intestinal cells during oxidative stress. The results of the current study suggest that carvacrol protection is closely related to anti-oxidative capacity, mitochondrial function, and autophagy inhibition (Figure 6). However, the mode of action of carvacrol in attenuating excessive autophagy, or possibly mitophagy, should be investigated in future studies.

Weaning stress presents significant challenges for piglets, primarily due to separation from the sow, dietary changes, and social stress from unfamiliar animals [45]. This stress leads to both structural and functional alterations in their intestines, impairing digestion and nutrient absorption and weakening the intestinal barrier, thus resulting in decreased feed intake, increased incidence of diarrhea, and slowed growth [15,21,45]. This study investigated the effect of carvacrol on the intestine during weaning stress utilizing an in vitro model. However, it is important to note that this in vitro model cannot fully replicate the complex microenvironment of the intestine during weaning stress, highlighting a significant limitation of the current study.

## 4. Materials and Methods

### 4.1. Chemicals and Reagents

Acridine orange, carvacrol, DMSO, MTT, and metformin hydrochloride were purchased from Sigma-Aldrich (St. Louis, MO, USA). The H_2_O_2_ solution was obtained from Honeywell (Seelze, Lower Saxony, Germany). DMEM/F12 and HBSS were purchased from GIBCO (Grand Island, NY, USA). FBS was purchased from HyClone (Logan, UT, USA). Antibodies against GAPDH (2118s), VDAC (4866), LC3B (2775), and p62 (5114s) were obtained from Cell Signaling Technology (Beverly, MA, USA). Antibodies against PINK1 (sc-33796), PARKIN (sc-30130), and β-actin (sc-47778) were obtained from Santa Cruz Biotechnology (Santa Cruz, CA, USA). Antibodies against OXPHOS (ab110411) were obtained from Abcam (Trumpington, Cambridge, UK). The HRP-conjugated secondary antibodies, anti-rabbit IgG (7074P2) and anti-mouse IgG (sc-2005), were purchased from Cell Signaling Technology (Beverly, MA, USA) and Santa Cruz Biotechnology (Santa Cruz, CA, USA), respectively.

### 4.2. Cell Culture and Treatment

IPEC-J2 cells were purchased from Leibniz Institute DSMZ (Braunschweig, Germany) and incubated in DMEM/F-12 with 10% FBS at 37 °C in a humidified incubator containing 5% CO_2_. The cells were passaged at 80% confluence, with passage numbers ranging from 10 to 25 used in this study.

After the IPEC-J2 cells were cultured for 24 h for proper attachment, they were treated with or without carvacrol for 6 h, and then H_2_O_2_ was added without changing the medium for 1 to 24 h. Carvacrol was diluted to 1 M in DMSO. For each treatment, the final concentration of DMSO was set at 0.05%. The indicated concentrations of H_2_O_2_ were freshly prepared in PBS and added to the culture medium. After treatment, the cells were harvested for further analysis.

### 4.3. Cell Viability Assay

Cell viability was measured through an MTT assay. IPEC-J2 cells were cultured in the presence or absence of carvacrol or H_2_O_2_. After treatment, the medium was renewed with 350 μL of DMEM/F12 containing 10% FBS, and 35 μL of 5 mg/mL MTT solution was added to each well. After culturing for 1.5 h, the supernatant was removed, and cells were resuspended with 200 μL DMSO to dissolve MTT formazan. The absorbance of the solution was measured at 570 nm and 690 nm using a spectrophotometer (BMG Labtech, Ortenberg, Germany). Cell viability was calculated by subtracting the absorbance at 690 nm from the absorbance at 570 nm and was represented as a percentage relative to the control.

### 4.4. Malondialdehyde (MDA) Level

The intracellular MDA level was used as a marker of lipid peroxidation. After treatment, IPEC-J2 cells were collected using trypsin and lysed using RIPA lysis buffer (Merck Millipore, Burlington, MA, USA) and centrifuged at 12,000× *g* for 30 min at 4 °C. The cell lysates (90 μL) were then mixed with 135 μL of thiobarbituric acid solution (0.1%) and 765 μL of trichloroacetic acid-HCl reagent (25% trichloroacetic acid/0.6N HCl = 5:2). After a water bath at 95 °C for 30 min, the mixture was cooled and centrifuged at 10,000× *g* for 3 min at 4 °C. The absorbance of the supernatant was measured at 535 nm using a spectrophotometer (BMG Labtech). MDA levels are expressed as nmol/mg of cellular protein.

### 4.5. Intracellular ROS Generation

Intracellular ROS levels were measured using a DCFDA/H2DCFDA—Cellular ROS Assay Kit (ab113851; Abcam, Cambridge, UK) according to the manufacturer’s instructions. IPEC-J2 cells were seeded in a 96-well black plate with a clear bottom (1.85 × 10^4^ cells/cm^2^) and treated with or without carvacrol for 6 h. At 45 min prior to H_2_O_2_ treatment, IPEC-J2 cells were stained with DCFDA at 37 °C in the dark. After H_2_O_2_ treatment, fluorescence excitation at 485 nm and emission at 535 nm were measured using a spectrophotometer (BMG Labtech).

### 4.6. Activities of Catalase and Superoxide Dismutase (SOD)

Catalase activity was determined using a CAT assay kit (707002; Cayman Chemical, Ann Arbor, MI, USA), according to the manufacturer’s instructions. In brief, after treatment, IPEC-J2 cells were collected in cold buffer (containing 50 mM potassium phosphate and 1 mM EDTA, pH 7.0) with cell scrapers, and centrifuged at 10,000× *g* for 15 min at 4 °C. The supernatant, assay buffer, and methanol were mixed. H_2_O_2_ was added to initiate the reaction and potassium hydroxide was added to stop the reaction. After the addition of Catalase Purpold for 10 min, potassium periodate was added and the absorbance at 540 nm was measured using a spectrophotometer (BMG Labtech). Catalase activity was represented as U/mg of cellular protein.

SOD activity was determined using a SOD assay kit (706002, Cayman Chemical), according to the manufacturer’s instructions. In brief, after treatment, IPEC-J2 cells were collected in cold HEPES buffer (containing 20 mM HEPES, 1 mM EGTA, 210 mM mannitol, and 70 mM sucrose, pH 7.2) with cell scrapers, and centrifuged at 1500× *g* for 5 min at 4 °C. The supernatant, Radical Detector, and xanthine oxidase were mixed and incubated on a shaker for 30 min at room temperature. The absorbance at 450 nm was measured using a spectrophotometer (BMG Labtech). SOD activity was represented as U/mg of cellular protein.

### 4.7. Mitochondrial ROS Detection

The mitochondrial ROS level was detected through MitoSOX^TM^ Red staining (M36008, Invitrogen, Grand Island, NY, USA). After treatment, the IPEC-J2 cells were stained with MitoSOX Red in HBSS. The fluorescence excitation at 500 nm and emission at 585 nm was measured continuously at 37 °C in the dark using a spectrophotometer (BMG Labtech).

For fluorescence imaging, cells were washed twice with warm PBS after treatment and then stained with MitoSOX Red in HBSS for 30 min at 37 °C in the dark. After removing the MitoSOX Red solution, the cells were stained with Hoechst (H3570; Invitrogen) in HBSS at room temperature in the dark. After two washes with warm HBSS, fluorescence imaging was performed using a Leica DM IL LED inverted microscope (Leica Microsystems, Wetzlar, Germany). The excitation and emission wavelengths for blue fluorescence were 350 nm and 460 nm, respectively, and those for red fluorescence were 540 nm and 605 nm, respectively. All images were obtained using the same parameters.

### 4.8. ATP Production

Intracellular ATP levels were assessed using an ATP colorimetric/fluorometric assay kit (K354-100; BioVision, Milpitas, CA, USA), according to the manufacturer’s instructions. In brief, after treatment, the cell lysates were deproteinized with a 10 kDa spin column and centrifuged at 12,000× *g* for 30 min at 4 °C. The deproteinized sample, assay buffer, and reaction mix were mixed for further reactions. Fluorescence excitation and emission were measured at 535 nm and 587 nm, respectively, using a spectrophotometer (BMG Labtech). ATP levels were represented as nmol/mg of cellular protein.

### 4.9. Quantification of Citrate Synthase Activity

Citrate synthase activity was determined using a citrate synthase assay kit (ab239712; Abcam), according to the manufacturer’s instructions. Briefly, after treatment, IPEC-J2 cells were centrifuged, and the supernatant, assay buffer, and reaction mix were combined for further reactions. The absorbance at 412 nm was continuously measured using a spectrophotometer (BMG Labtech). Citrate synthase was represented as U/mg of cellular protein.

### 4.10. Western Blotting

IPEC-J2 cells were lysed with RIPA lysis buffer and protein concentrations were determined using the Dual-Range^TM^ Bradford reagent (BR05-500, Visual Protein Biotech, Taipei, Taiwan). The total protein (10–20 μg) was loaded per sample per lane and separated by SDS-PAGE. After electrophoretic transfer to a polyvinylidene difluoride membrane (Merck Millipore, Burlington, MA, USA), the membrane was blocked with TBS-T (20 mM Tris, 150 mM NaCl, and 0.1% Tween-20) containing 5% skim milk. Then, the membrane was incubated in one of the following primary antibodies at 4 °C overnight: anti-GAPDH (1:10,000), anti-VDAC (1:1000), anti-LC3B (1:1000), anti-p62 (1:1000), anti-OXPHOS (1:1000), anti-PINK1 (1:1000), anti-PARKIN (1:1000), and anti-β-actin (1:1000). After three washes with TBS-T, the membrane was incubated with an HRP-conjugated secondary antibody, anti-rabbit IgG (1:20,000), or anti-mouse IgG (1:20,000) individually. Immunoblotting was performed using a chemiluminescent HRP substrate (Merck Millipore, Burlington, MA, USA), according to the manufacturer’s instructions. Chemiluminescence was detected using the ChemiDoc Imaging System (17001401, Bio-Rad, Hercules, CA, USA) and quantified using ImageLab Software 6.1 (Bio-Rad, Hercules, CA, USA).

### 4.11. Fluorescence Imaging of Acidic Vacuoles

Acridine orange staining was performed to visualize the intracellular acidic vacuoles (autolysosomes and lysosomes) as autophagy markers. After treatment, IPEC-J2 cells were stained with 1 μg/mL acridine orange in culture medium at 37 °C in an incubator for 15 min and then washed three times with PBS. Fluorescence imaging was performed using the Leica DM IL LED inverted microscope (Leica Microsystems). The excitation and emission wavelengths for green fluorescence were 480 nm and 535 nm, respectively, and those for red fluorescence were 540 nm and 605 nm, respectively. All images were obtained using the same parameters.

### 4.12. Detection of Apoptotic and Necrotic Cells

Apoptotic and necrotic cells were detected using a PE Annexin V Apoptosis Detection Kit with 7-AAD (640934; BioLegend, San Diego, CA, USA), according to the manufacturer’s instructions. After treatment, IPEC-J2 cells were collected using trypsinization and double-stained with Annexin V and 7-AAD. Then, cells were washed with cold cell staining buffer (PBS containing 2% heat-inactivated FBS and 0.09% sodium azide) and resuspended in a binding buffer. Data were analyzed with the BD FACSAria^TM^ III Cell Sorter (BD Biosciences, San Jose, CA, USA) within 1 h. For cells negative for Annexin V and 7-AAD, they were recognized as live cells. Cells positive for Annexin V and negative for 7-AAD were recognized as early apoptotic cells. Cells positive for both Annexin V and 7-AAD were recognized as late apoptotic or necrotic cells.

### 4.13. Statistical Analysis

Values are expressed as means ± SEM. Statistical analysis was conducted using one-way ANOVA followed by Tukey’s multiple comparisons test. Differences were considered statistically significant at *p* < 0.05. All statistical analyses were performed using Prism 9.0 (GraphPad, Boston, MA, USA).

## 5. Conclusions

This study demonstrated that carvacrol effectively ameliorated cell death, oxidative stress, and mitochondrial dysfunction in porcine intestinal epithelial cells during H_2_O_2_-induced oxidative damage. Furthermore, our study is the first to show that autophagy inhibition is indispensable for carvacrol’s protection of porcine intestinal epithelial cells during oxidative stress.

## Data Availability

The original contributions presented in this study are included in the article. Further inquiries can be directed to the corresponding author.
